# Mayaro Virus Infects Human Chondrocytes and Induces the Expression of Arthritis-Related Genes Associated with Joint Degradation

**DOI:** 10.3390/v11090797

**Published:** 2019-08-29

**Authors:** Michèle Bengue, Pauline Ferraris, Cécile Baronti, Cheikh Tidiane Diagne, Loïc Talignani, Sineewanlaya Wichit, Florian Liegeois, Catherine Bisbal, Antoine Nougairède, Dorothée Missé

**Affiliations:** 1 MIVEGEC, IRD, Univ. Montpellier, CNRS, 34394 Montpellier, France; 2 Unité des virus émergents, Aix Marseille Univ-IRD 190, Inserm 1207-IHU Méditerranée Infection, 13385 Marseille, France; 3 Department of Clinical Microbiology and Applied Technology, Faculty of Medical Technology, Mahidol University, Nakhon Pathom 73170, Thailand; 4 PhyMedExp, CNRS UMR 9214, INSERM U1046, University of Montpellier, 34295 Montpellier, France

**Keywords:** arbovirus, Mayaro virus, matrix metalloproteinases, extracellular matrix, chondrocyte, synoviocyte, osteoblast, innate immunity, IL-6, TNF-α

## Abstract

Mayaro virus (MAYV) is an emerging arthritogenic alphavirus belonging to the Togaviridae family. Infection leads to a dengue-like illness accompanied by severe polyarthralgia. However, the molecular and cellular mechanisms of arthritis as a result of MAYV infection remain poorly understood. In the present study, we assess the susceptibility of human chondrocytes (HC), fibroblast-like synoviocytes and osteoblasts that are the major cell types involved in osteoarthritis, to infection with MAYV. We show that these cells are highly permissive to MAYV infection and that viral RNA copy number and viral titers increase over time in infected cells. Knowing that HC are the primary cells in articular cartilage and are essential for maintaining the cartilaginous matrix, gene expression studies were conducted in MAYV-infected primary HC using polymerase chain reaction (PCR) arrays. The infection of the latter cells resulted in an induction in the expression of several matrix metalloproteinases (MMP) including MMP1, MMP7, MMP8, MMP10, MMP13, MMP14 and MMP15 which could be involved in the destruction of articular cartilage. Infected HC were also found to express significantly increased levels of various IFN-stimulated genes and arthritogenic mediators such as TNF-α and IL-6. In conclusion, MAYV-infected primary HC overexpress arthritis-related genes, which may contribute to joint degradation and pathogenesis.

## 1. Introduction

Mayaro virus (MAYV) is a little-known emerging mosquito-borne virus of the *Togaviridae* family which include Chikungunya (CHIKV), Ross River (RRV), Sindbis and Una viruses. The virus was first isolated in Trinidad and Tobago in 1954 from the serum of febrile forest patients [1]. MAYV is phylogenetically related to the Semliki forest antigenic complex and is transmitted by Haemagogus mosquitoes belonging to the *Aedes* species, including *aegypti, albopictus* and *scapularis* [2]. In the past, small and occasional outbreaks were reported in the Amazon basin of Brazil, as well as in other areas in South America [3,4]. Mayaro fever has recently become an important public health problem in Brazil after several cases of this disease were reported in the region [5]. There have also been recent reports of Mayaro fever in other areas of South America and Central America [1]. Its recent appearance in the Caribbean (Haïti) and the increase of imported cases from South America to other parts of the world indicate that MAYV may be extending its reach. The MAYV case reported in Haiti may be an indication that the virus could potentially spread to the southern areas of the United States, especially due to the increase of immigration and tourism in the area. Both regions also have similar climates and mosquito vector species that could favor the distribution of the virus. Like other alphaviruses, such a Ross River virus and Onyong-nyong virus, MAYV consists of a positive single-stranded RNA of approximately 12 Kb encoding two polyproteins, which consist of four non-structural proteins (nsP1, nsP2, nsP3, nsP4) and five structural proteins (C, E1, E2, E3, 6k) [2]. Phylogenetic studies that used whole-genome sequencing have classified MAYV strains into two major genotypes: genotype D, widely distributed in the Pan-Amazonia, and L, originating from Belterra, Brazil, and a third minor genotype, N (new), consisting of a single sequence isolated from Peru in 2010 [6]. MAYV causes a self-limited illness characterized by fever, headache, vomiting, diarrhea, muscle pain, rash and long-lasting arthralgia—symptoms similar to those induced by other arboviruses such as CHIKV. Since testing for MAYV is not systematic, the number of cases of patients infected by this virus may be underestimated. To date, there are no available antiviral drugs or vaccines against MAYV infection. Because MAYV has received far less attention than other arthritogenic alphaviruses, such as CHIKV, the pathogenesis of MAYV infection still remains poorly understood. Infection leads to a dengue-like illness accompanied by severe polyarthralgia [7], however, the molecular and cellular mechanisms of arthritis due to MAYV remain poorly understood. Moreover, the signaling pathways and antiviral immune response of the host elicited by this virus remain to be determined. To develop effective treatments and vaccines, it is important to understand the mechanisms of the host immune response. Infection by arthritogenic alphaviruses results in severe inflammation in bone, joint and muscle tissues [8,9,10,11,12,13]. Several studies describe the persistence of joint pain for months or even years and indicate that the pathogenesis involved in alphavirus-induced joint damage is determined by virus persistence and virulence as well as by host immune responses [9,14,15,16,17,18]. It has been reported that 50% of MAYV infected individuals develop persistent arthralgia affecting the joints [7]. Using murine models of alphavirus-induced arthritis, cells from musculoskeletal tissues have been described as targets for alphavirus replication [10,19,20,21]. The aim of the present study was to analyze whether the main cells involved in bone and cartilage structure and formation such as human chondrocytes (HC), fibroblast-like synoviocytes (HFLS) and osteoblasts (HOB) are permissive to MAYV infection. We also determined the nature of the cellular response induced by MAYV in primary HC which are responsible for cartilaginous matrix composition and integrity. 

## 2. Materials and Methods

### 2.1. Virus and Cell Lines

We used the *Homo sapiens*/Haiti-1/2015 MAYV strain (Genotype L; GenBank accession number KX496990) [22], a low-passaged strain isolated in 2015 from the plasma of an 8-year-old Haitian child who had fever and abdominal pain. This strain derived from a reverse genetics system based on its GenBank sequence. The infectious subgenomic amplicons (ISA) method was used to rescue infectious viral particles as previously described [23,24]. Briefly, the whole viral genome was de novo synthesized in three double-stranded overlapping DNA fragments. First and last DNA fragments were flanked respectively at the 5′ and 3′ termini by the human cytomegalovirus immediate early enhancer/promoter and the hepatitis delta ribozyme followed by the simian virus 40 polyadenylation signal. These synthetic DNA fragments were used as template to generate subgenomic amplicons by polymerase chain reaction (PCR). An equimolar mix of the three purified subgenomic amplicons was used for cell transfection (HEK-293 cells, Lipofectamine 3000, Thermo Fisher Scientific, Illkirch-Graffenstaden, France). Culture medium containing infectious cell supernatant was then serially passaged three times in Vero-E6 cells. This cell line was also used for viral titration and grown in Dulbecco’s modified Eagle’s medium (DMEM; Invitrogen, Cergy Pontoise, France), supplemented with 5% FCS; Lonza, Basel, Switzerland).

### 2.2. Primary Human Cells 

Primary HFLS, HOB and HC were obtained from Sigma (Saint Quentin Fallavier, France) and cultured in synoviocyte growth medium, osteoblast growth medium and chondrocyte growth medium (Sigma, Saint Quentin Fallavier, France), respectively, at 37 °C in a 5% CO_2_ humidified incubator. 

### 2.3. Infection of Human Fibroblast-Like Synoviocytes (HFLS), Human Osteoblasts (HOB) and Human Chondrocytes (HC)

Monolayer cultures (2.5 × 10^5^ cells) of each cell types were washed once with Hank’s balanced salt solution (HBSS) and inoculated with MAYV at a multiplicity of infection (MOI) of 1, at 37 °C for 2 h with gentle agitation. The inoculum was then removed and cells were washed twice with HBSS. One mL of the corresponding culture medium was added and cells were kept at 37 °C in a 5% CO_2_ atmosphere. Aliquots of 1 mL of supernatant fluid was harvested at the indicated time points post-infection.

### 2.4. Virus Titration

The harvested culture supernatants were used in a plaque assay for determination of the efficiency of viral replication. Briefly, 10-fold dilutions of purified virus were spread onto monolayers of Vero-E6 cells (1.5 × 10^5^ cells/well) in 24-well tissue-culture trays at 37 °C for 2 h preceding infection. A mix of nutriment solution with agar was then added. At 5 days post infection (dpi), the cells were fixed with 3.7% paraformaldehyde for 15 min and stained with the crystal violet solution in 20% ethanol (1%) at room temperature for 1 h. Viral titers were calculated by observation of plaque formation. Mock-infected cells, incubated with the culture supernatant from uninfected C6/36 cells were considered as controls.

### 2.5. Quantification of Mayaro Virus (MAYV) by Real-Time Polymerase Chain Reaction (PCR) 

According to the manufacturer’s protocol, viral RNA was extracted from infected primary cells by using Tri reagent (Sigma, Saint Quentin Fallavier, France). The RNA pellet was stored at −80 °C after it was resuspended in 30 µL of RNase-free distilled water. Moloney murine leukemia virus (M-MLV) reverse transcriptase (Promega, Charbonnieres, France), was used to conduct reverse transcription of one μg of RNA. Real-time PCR was performed with Maxima probe/ROX quantitative PCR (qPCR) master mix (Fermentas, Saint Remy les Chevreuses, France). Each 25 µl reaction mixture contained a final concentration of 500 nM forward primer, 500 nM reverse primer, 250 nM specific probe, and 1× (final concentration) Maxima probe/ROX qPCR master mix. The quantification of MAYV were conducted using the following primers and probes: MAYV_F TGCGCCTGCCAGGAGAATGCTGT; MAYV_R TCGCCTGATGCCTTGGCCAACT; FAM-ACGTACCATTGTGTGTGCCCAAT-BHQ. 

MAYV was amplified through activation at 95 °C for 10 min followed by 40 amplification cycles of 95 °C for 15 s, 60 °C for 30 s, and 72 °C for 30 s, using Applied Biosystems 7300 real-time PCR system. To quantify viral RNA, each sample’s threshold cycle (*C_T_*) value was compared with a MAYV RNA standard curve which was obtained in three steps. First, total viral RNA from the cell culture was purified using a QIAamp viral RNAkit (Qiagen, Courtaboeuf, France) following the manufacturer’s protocol. Second, a standard RT-PCR was carried out by using the following primers: (T7-MAYV_F; TAATACGACTCACTATAGGGTGCGCCTGCCAGGAGAATGCTGT and MAYV_R, TCGCCTGATGCCTTGGCCAACT) for MAYV. Third, the PCR product was used to generate MAYV RNA fragments by in vitro transcription using a MAXIscript kit (Ambion, Austin, TX). The RNA strands generated were determined by spectrophotometry and converted to numbers of molecular copies by using the following formula: number of *y* molecules per microliter = [(*x* grams per microliter of RNA)/(transcript length in base pairs × 340)] × 6.02 × 10^23^. 

### 2.6. RT2 Profiler PCR Array

The RT2 First Strand kit (Qiagen, Valencia, CA, USA) was used for the synthesis of the complementary DNA strand using 400 µg of total RNA from samples extracted with Tri reagent (Sigma, Saint Quentin Fallavier, France), following the manufacturer instructions RNA concentrations were determined using a NanoDrop spectrophotometer. RT^2^ Profiler™ PCR Array Human Extracellular Matrix and Adhesion Molecules (PAHS-013Z) and RT^2^ Profiler™ PCR Array Human Antiviral Response (PAHS-122Z) were used in the present study. A 384 well plate was used in which each group of 4 wells contains the primer of a distinct gene. Five housekeeping genes (β-actin (ACTB), β-2-microglobulin (B2M), glyceraldehyde-3-phosphate dehydrogenase (GAPDH), hypoxanthine phosphoribosyltransferase1 (HPRT1) and ribosomal protein, large, P0 (HPLP0) were used as internal control. The Roche LightCycler 480 real-time cycler was used to amplify the DNA with a thermal cycling of 95 °C for 10 min followed by 40 cycles of 95 °C for 15 s and 60 °C for 1 min, followed by a melting curve acquisition step.

Briefly, cDNA reaction mixture (20 µL per sample) was diluted in 91 µL of nuclease-free H2O (Qiagen), 102 μL aliquot was mixed with 548 µL of RNase free H2O and 650 μL of 2 × RT2 SYBR green RT2 master mix following the manufacturer’s instructions, and 10 µL of the mix was loaded into each well. The average of reverse transcription control values and positive PCR control *CT* values was used to normalize gene expression and determine fold changes between groups. Changes in mRNA expression were analyzed using the ΔΔCt method. The experiments were performed in biological and technical triplicates with which the confidence level was determined. Target gene Ct values were normalized with housekeeping genes. Gene modulation was considered statistically significant at a 95% confidence level (*p* < 0.05). The confidence level was determined from data obtained in triplicate for each sample. RT^2^ profiler RT-PCR array data analysis software (version 3.5) was used for statistical analysis.

### 2.7. Gene Expression Analysis by Real-Time PCR

The total RNA 1 μg of Human chondrocyte cells was used to synthesize cDNA using the MMLV reverse transcription Kit (Promega, Charboniere, France). Real-time PCR was performed using 1 μL of cDNA with specific primers targeting the genes of interest. For the expression of each gene, a reaction volume of 9 μL contained a primer concentration of 400 nM and 4 μL of Fast Eva Green Master Mix (Invitrogen; Thermo Fisher Scientific, Inc.). The cycling conditions were 45 cycles of 95 °C for 15 s, 60 °C for 15 s, and 72 °C for 20 s. The transcript level (fold change) was quantified by calculating the 2^−ΔΔ*CT*^ value, with glyceraldehyde-3-phosphate dehydrogenase (GAPDH) mRNA as an endogenous control. The primers used in the present study are listed in a Supplementary Table S1.

### 2.8. Measurements of Cytokines

Concentrations of IL-6 and TNF-α in the culture supernatants of HC were determined using enzyme-linked immunosorbent assay (ELISA) kits (Quantikine, R&D Systems, Mineapolis, MN, USA) according to the manufacturer’s instructions. The absorbance at 450 nm was measured using a microplate reader (Infinite M200PRO TECAN, Männedorf, Switzerland) and concentrations of cytokines were determined by interpolation of a standard curve.

### 2.9. Western Blot

Cells were lysed in RIPA buffer (150 mM NaCl, 5 mM β-mercaptoethanol, 1% NP-40, 0.1% sodium dodecyl sulfate, 50 mM Tris-HCl, pH 8) supplemented with protease and phosphatase inhibitor cocktail solution (Sigma, Saint Quentin Fallavier, France). The protein concentration was determined using bicinchoninic acid assay quantification kit (Thermo Scientific, Saint Herblain, France) following the manufacturer’s procedure. Equal amounts of proteins were mixed with Laemmli sample buffer and subjected to sodium dodecyl sulfate polyacrylamide gel electrophoresis (SDS-PAGE) and electrotransferred onto a nitrocellulose membrane. The membrane was blocked with phosphate-buffered saline (PBS) 0.05% Tween 20 containing 5% skim milk and incubated overnight with primary antibodies. The following primary antibodies were used: anti-Viperin, anti-MMP9 anti-MMP2, anti-MMP14, and anti-β-actin were obtained from Cell Signaling (Cell Signaling Technology, Inc., Danvers, MA, USA). The membranes were washed 3 times with PBS-Tween and incubated for 2 h at room temperature with horseradish peroxidase-coupled secondary antibodies (Sigma). Proteins were detected by chemiluminescence using a SuperSignal West Pico chemiluminescent substrate kit (Thermo Scientific). 

### 2.10. Cell Viability Assay

ReadyProbes® Cell Viability Imaging Kit (Blue/Green) (Thermo Fisher Scientific, Illkirch-Graffenstaden, France) was used to determine the viability of HC cells following the manufacturer’s recommendation. Briefly, HC were inoculated with MAYV at the MOI of 1, at 6, 24 and 48 h post-infection (hpi), 2 drops of NucBlue® Live reagent (Hoechst 33342) and NucGreen® Dead reagent were added into each wells (2 drops/ mL of cell growth media) at room temperature for 5min. Cell viability was determined by counting total cells and dead cells: NucBlue® Live reagent stained the nuclei of live cells and were detected with a DAPI filter; NucGreen® Dead reagent stained the nuclei of dead cells and were detected with standard GFP (green) filter by fluorescence microscopy using a Leica microscope at 10-fold magnification.

## 3. Results and Discussion

**Figure 1:**
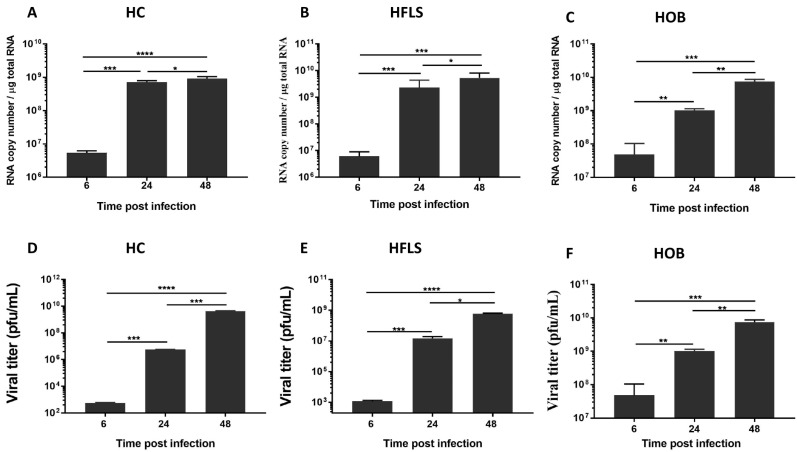
Human chondrocytes (HC), fibroblast-like synoviocytes (HFLS) and osteoblasts (HOB) are permissive to Mayaro virus (MAYV) infection. Cells were infected with MAYV at a multiplicity of infection (MOI) of 1. Viral RNA expression was then measured at 6, 24 and 48 h post-infection using real-time polymerase chain reaction (PCR) (**A**–**C**). Plaque assay analysis of culture supernatants of MAYV-infected cells were used to detect infectious viral particles (**D**–**F**). Experiments were performed three times each in triplicate (error bars represent standard errors of the means [SEM]). Comparisons between the indicated time point post infection were performed using one-way analysis of variance (ANOVA) and Tukey’s multiple comparisons test with a *P*-value significant when * *p* < 0.05, ** *p* < 0.01, *** *p* < 0.001, **** *p* < 0.0001.

Severe join pain and stiffness are the hallmark of arthritogenic alphaviruses [25,26]. In order to determine the permissiveness of primary HC, HOB and HFLS, cells were infected with MAYV at a multiplicity of infection (MOI) of 1. We first quantified the intracellular viral RNA by real-time PCR at different hours post-infection (hpi). The amounts of viral transcripts were markedly high in all cell types reaching 10^8^–10^9^ RNA copies/μg at 48 hpi and could be determined as soon as 6 hpi. The results show a gradual increase in viral RNA copy numbers during the course of infection (Figure 1A–C). Next, we evaluated the ability of the infected cells to produce viral progeny by determining viral titers in the supernatants of MAYV-infected cells, using a plaque assay. The production of viral particles increases over time, indicating active viral replication in the infected cells (Figure 1D–F). These findings demonstrate that primary HC, HOB and HFLS are susceptible to MAYV infection. These results are in accordance with previous studies demonstrating that bone cells can be productively infected by arthritogenic alphaviruses such as RVV and CHIKV [19,20,27]. 

**Figure 2:**
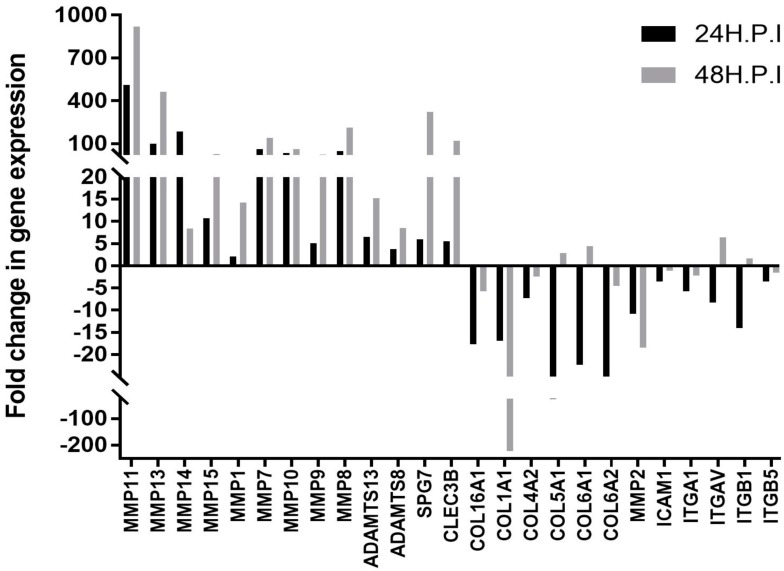
Modulation of extracellular matrix genes by MAYV in primary human chondrocytes. Human chondrocytes were infected with MAYV at MOI 1. The modulation of extracellular matrix and adhesion molecules gene expression was quantified by RT^2^ Profiler PCR arrays at 6 and 24 h post-infection. The graph represents the most important expressed genes. Gene modulation was considered statistically significant at a 95% confidence level (*p* < 0.05). RT^2^ profiler real-time PCR (RT-PCR) array data analysis software (version 3.5) was used for statistical analysis.

Articular cartilage is a tissue mainly composed of chondrocytes, enclosed in an extracellular matrix (ECM) [28]. These cells are responsible for the synthesis and maintenance of all ECM molecules [28,29,30], thereby conferring to cartilage its functions of mechanical support and joint lubrification. In order to evaluate the impact of MAYV on the expression of genes involved in the composition of the ECM, primary HC were infected with MAYV, at a MOI of 1, at 24 and 48 hpi and mRNA expression of 84 genes important for cell–cell and cell–matrix interactions were quantified using a specific qPCR array analysis (Figure 2 and Table S2). The infection of these cells resulted in an early (24 hpi) upregulation of several matrix metalloproteinases (MMPs) including MMP1, MMP7, MMP8, MMP9, MMP10, MMP11, MMP13 (Figure 2 and Table S2). Interestingly, a substantial cartilage erosion with overproduction of matrix degrading enzymes such as MMP9 has previously been reported in the joints of RRV-infected-mice [31]. Increased level of MMP 9 protein expression was validated by Western blotting analysis (Figure S2). MMPs play a critical role in the destruction of articular cartilage in disease processes such as arthritis [32] and, therefore, could contribute to bone degradation during MAYV infection. The gene expression levels of the Membrane Type 1 MMP (MMP14) and Type 2 MMP (MMP15) were dramatically increase at 24 hpi in MAYV-infected cells, with a fold change of 183 and 10 respectively, as compared to mock-infected cells (Figure 2 and Table S2). These enzymes are well-documented for their role in pericellular collagenolysis [33]. The expression levels of seven MMPs were validated by individual PCR using different primer sets and different chemistry. As expected, apart from MMP2 expression the transcript levels of MMP1, MMP8, MMP10, MMP13, MMP14 and MMP15 were upregulated following MAYV infection (Figure S1). 

**Figure 3:**
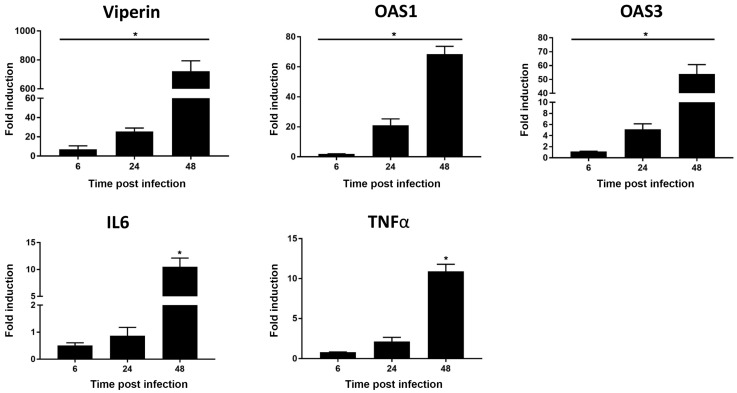
Regulation of immune genes in MAYV-infected human chondrocytes. Primary HC were exposed to MAYV at a MOI of 1, and mRNA levels of the corresponding genes were quantified by real-time PCR. Fold change compared to mock-infected cells are shown. Data are representative of three independent experiments, each performed in triplicate (error bars represent SEM). Differences between data were analyzed using the Wilcoxon–Mann–Whitney test. *p* values of < 0.05 were considered significant (*).

Interestingly, genes encoding collagens Col16A1, Col1A1, Col4A2, Col5A1, Col6A1 and Col6A2 were strongly downregulated at 24 hpi. It would be of interest to evaluate whether there is a link between the overexpression of MMPs transcripts and the subsequent decreased expression of these ECM structural components. A recent study showed that expression of type I and type II collagen was downregulated upon infection of HC by RVV [20], corroborating our results. Transcript levels of the other ECM proteases such as ADAMTS8, ADAMTS13 and SPG7 were also increased in HC exposed to MAYV (Figure 2 and Supplementary Table S2). CLEC3B has previously been identified as a gene candidate associated with osteoarthritis (OA) [34,35]. Expression levels of CLEC3B transcripts was found to be upregulated in MAYV-infected HC. In contrast, gene expression levels of the cell–cell adhesion molecules ICAM1, ITGA1, ITGAV, ITGB1, and ITGB5 were downregulated at 24 hpi in infected primary HC (Figure 2 and Supplementary Table S2). These results indicate that MAYV strongly modulates the gene expression levels of human extracellular matrix and adhesion molecules in the latter cells. Next, we evaluated whether MAYV induces an innate antiviral immune response in HC. For this purpose, the antiviral gene expression profile in infected cells were quantified at 24 and 48 hpi using a human qPCR array covering 84 human antiviral genes (Table 1). This comparative analysis with mock-infected cells showed that the induction of the antiviral response by HC increased in a time dependent-manner. MAYV induced the transcription of the pattern recognition receptors TLR3, RIG-I, and MDA5 which are known to recognize several arboviruses [36,37]. The detection of MAYV by PRRs increased the mRNA expression of the transcription factor IRF7 leading to the strongly enhanced *interferon-β* gene expression, as well as several interferon-stimulated genes (ISG), including *ISG15, MX1, OAS2*. Individual quantitative RT-PCR analyses also demonstrated that MAYV induced, in a dose dependent-manner, the transcript levels of other ISG which have been shown to exert antiviral effect against CHIKV [38,39]: *Viperin, OAS1,* and *OAS3* (Figure 3). Protein levels of Viperin were also increased upon exposure of HC with MAYV (Supplementary Figure S2). It was shown previouly that Viperin is critical for the control of CHIKV infection and joint pathology [40]. In fact, mice lacking *Viperin* had higher viremia and severe joint inflammation. It would be interesting to explore whether there is a link between the level of Viperin and expression of ECM components following MAYV infection.

**Figure 4:**
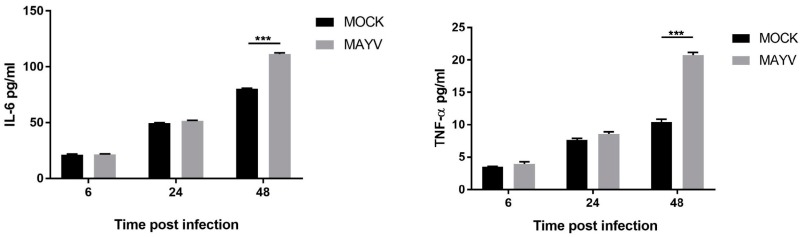
MAYV increases the production of proinflammatory cytokines in primary human chondrocytes. IL-6 and TNF-α secretion were quantified in the supernatant of infected cells using ELISA. Mock-infected cells were used as control. The data represent three independent experiments, each performed in triplicate. The two-way ANOVA statistical analysis with Bonferroni’s test was used to analyse the set of data. *** *p* < 0.0001.

The data also revealed that mRNA expression of the arthritogenic mediators TNF-α and IL-6 were significantly increased at 48 hpi (Figure 3). We determined the concentration of IL-6 and TNF-α in the culture supernatants of MAYV-infected cells and found upregulation of both cytokines at 48 hpi (Figure 4). Interestingly, it has been shown that these pro-inflammatory cytokines are associated with OA occurrence and are able to activate the mitogen-activated protein kinase and Janus kinase/signal transducers and activators of transcription pathways by binding the cytokine to their receptors on chondrocytes and synoviocytes, leading to increased synthesis of MMPs and ADAMTS [41,42,43]. A longitudinal study assessing the immune response following MAYV infection showed that TNF-α remained significantly elevated in patients who developed persistent arthralgia when compared with healthy donors [13]. IL-6 has been previously identified as predictor of disease severity in longitudinal studies assessing the inflammatory response following CHIKV infection [44,45]. In agreement with our results infection of HC by RVV also induces the production of TNF-α and IL-6 [20].

**Figure 5:**
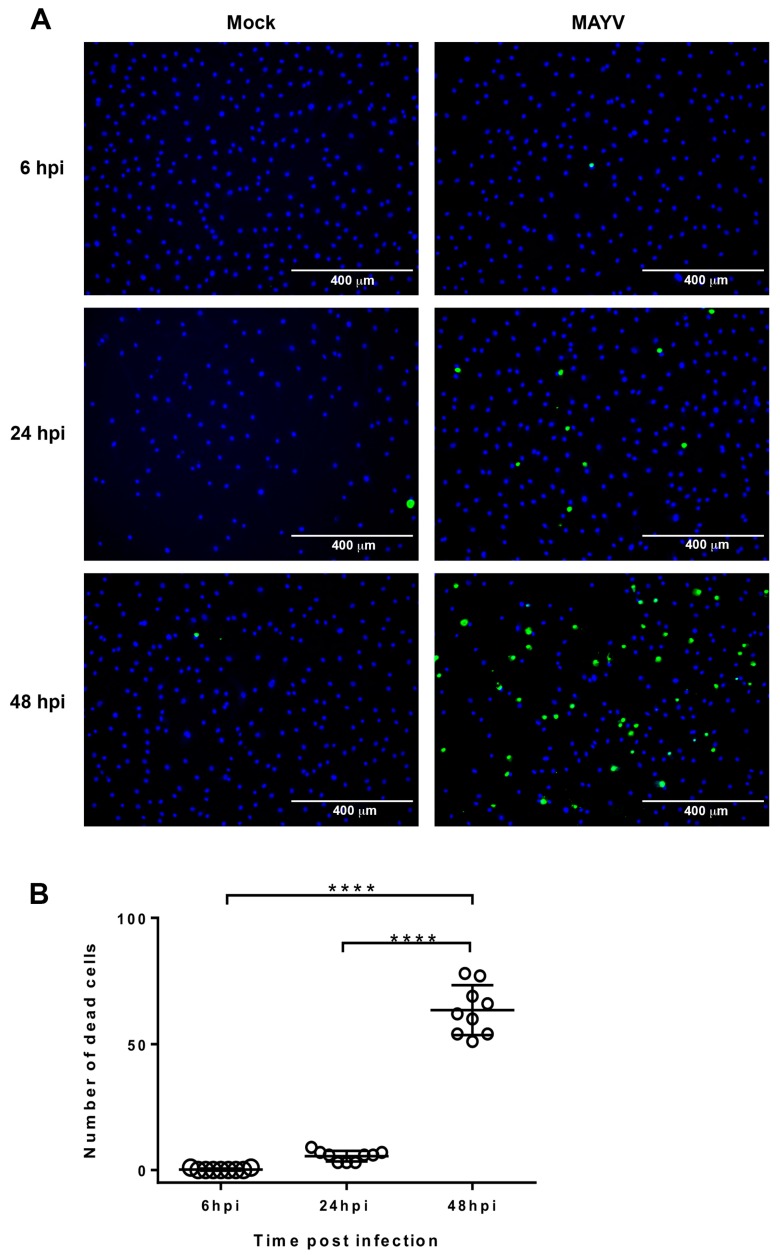
MAYV induces chondrocyte cell death. Primary HC were infected with MAYV at a MOI of 1. (**A**) The cell viability was determined at 6, 24 and 48 hpi and visualized with a fluorescence microscope. NucBlue® Live and NucGreen® Dead reagents were added to cells in full growth media and cell were visualized under fluorescent microscope. Mock-infected cells were used as control. (**B**) Median numbers of dead cells (green fluorescent cells) counted in the fields of view at the indicated time points post infection. Each dot represents the number of cells counted in one field. Horizontal bars indicate the median. Comparisons of dead cell numbers were performed using one-way ANOVA (*p* < 0.0001) followed by Tukey’s post hoc test for multiple comparisons **** *p* < 0.0001. The data are representative of three independent experiments.

Finally, we evaluated whether MAYV was able to induce a cytopathic effect in HC. For this purpose, cells were infected with the virus at a MOI of 1 and cell viability were determined at different time post-infection by immunofluorescence staining the nuclei of dead cells with compromised plasma membranes (Figure 5A). Results showed that at 24 and 48 hpi, 8% and 30%, respectively, of cells were stained in MAYV-infected HC, as compared to mock-infected cells (Figure 5B), thereby demonstrating the capability of MAYV to induce chondrocyte cell death. Several studies demonstrated that apoptosis occurred more frequently in cartilage from osteoarthritis patients than in cartilage from healthy subjects [46] and that there was a positive correlation between the number of apoptotic chondrocytes and cartilage degradation [42]. Chondrocyte cell death was also linked to the overproduction of matrix degradative enzymes [47]. Nevertheless, further studies are needed to understand whether cell death is due to a bystander effect or direct cytopathic effects.

In conclusion, we show that primary HC, HOB and HFLS, the major cell types involved in arthritis, are permissive to MAYV infection and that HC may play a crucial role in the pathogenesis of the disease by overexpressing arthritogenic mediators and arthritis-related genes. 

## Figures and Tables

**Table 1:** Modulation of antiviral genes by Mayaro virus in primary human chondrocytes.

	Fold Change in Gene Expression at Indicated Time Postinfection ^a^				Fold Change in Gene Expression at Indicated Time Postinfection ^a^	
Gene	24 h	48 h		Gene	24 h	48 h
*AIM2*	−1.11	**15.85**		*IRF3*	−1.41	−1.14
***APOBEC3G***	1.37	**5.35**		*IRF5*	−1.05	−1.66
*ATG5*	−1.44	−1.58		***IRF7***	**9.04**	**24.08**
***AZI2***	−1.93	**−2.00**		***ISG15***	**30.84**	**176.88**
*CARD9*	1.38	−1.00		*JUN*	−1.15	1.11
*CASP1*	1.16	1.90		*MAP2K1*	1.10	1.08
***CASP10***	**2.31**	−1.07		*MAP2K3*	−1.61	−1.79
*CASP8*	−1.50	−1.51		***MAP3K1***	−1.66	**−2.44**
*CCL3*	1.87	**4.42**		*MAP3K7*	−1.80	−1.78
***CCL5***	**3.79**	**44.84**		*MAPK1*	−1.64	−1.54
*CD40*	−1.54	−1.09		*MAPK14*	−1.68	−1.49
*CD80*	−1.32	−1.73		*MAPK3*	−1.16	−1.94
*CD86*	−1.29	−1.00		*MAPK8*	−1.24	−1.84
*CHUK*	−1.10	1.05		*MAVS*	−1.99	−1.22
***CTSB***	1.06	**−3.50**		*MEFV*	1.34	−1.52
*CTSL1*	−1.53	1.06		***MX1***	**36.17**	**43.51**
***CTSS***	−1.3	**2.91**		***MYD88***	1.43	**2.71**
***CXCL10***	**25.28**	**304.44**		*NFKB1*	1.17	1.39
***CXCL11***	**18,25**	**242.19**		***NFKBIA***	−1.11	**2.17**
*CXCL9*	1.25	1.77		***NLRP3***	**2.73**	1.03
*CYLD*	−1.49	−1.01		*NOD2*	1.25	−1.76
*DAK*	−1.12	−1.53		***OAS2***	**13.06**	**28.18**
***DDX3X***	−1.57	**−2.05**		*PIN1*	1.24	1.27
***DDX58 (RIG–I)***	**6.68**	**13.12**		***PSTPIP1***	−1.65	**−2.44**
***DHX58 (LGP2)***	**2.22**	**6.42**		*PYCARD*	−1.73	−1.58
*FADD*	−1.21	−1.68		*PYDC1*	1.37	1.22
***FOS***	**15.42**	**9.00**		*RELA*	−1.01	1.41
*HSP90AA1*	−1.38	−1.03		***RIPK1***	1.44	**2.21**
***IFIH1 (MDA5)***	**6.51**	**16.34**		***SPP1***	−1.49	**2.13**
*IFNA1*	1.12	1.55		***STAT1***	1.84	**2.15**
***IFNA2***	**3.59**	**4.79**		*SUGT1*	−1.84	−1.61
***IFNAR1***	−1.54	**−2.11**		***TBK1***	**−2.36**	−1.65
***IFNB1***	**45.68**	**570.73**		*TICAM1*	−1.30	1.52
*IKBKB*	−1.26	−1.14		*TLR3*	1.14	**2.51**
*IL12A*	−1.64	−1.51		*TLR7*	1.16	1.03
*IL12B*	−1.24	1.12		*TLR8*	1.04	1.52
*IL15*	1.15	1.59		*TLR9*	−1.39	−1.10
***IL18***	**−2.09**	**−2.78**		*TNF*	ND	ND
***IL1B***	**2.02**	**6.71**		*TRADD*	1.27	1.30
***IL6***	**−2.24**	**2.24**		*TRAF3*	−1.13	−1.63
***IL8***	−1.37	**6.02**		***TRAF6***	**−2.66**	−1.77
*IRAK1*	1.84	−1.16		***TRIM25***	**2.08**	1.78

^a^ Values represent fold inductions of mRNA copy numbers in infected cells relative to mock-infected cells. Values in bold indicate differentially expressed genes. ND: not determined.

## Supplementary Materials

The following are available online at https://www.mdpi.com/1999-4915/11/9/797/s1: Figure S1: Mayaro virus induces upregulation of extracellular matrix breakdown genes, Figure S2: Expression of Viperin and MMP proteins following MAYV infection of primary human chondrocytes, Table S1. Primers used in this study, Table S2. Modulation of extracellular matrix genes by MAYV in primary human chondrocytes.
