# Supplementary material for: Mayaro Virus Infects Human Chondrocytes and Induces the Expression of Arthritis-Related Genes Associated with Joint Degradation
Source: Viruses. 2019 Aug 29;11(9):797. doi: 10.3390/v11090797 (PMC6783875; doi:10.3390/v11090797)
Supplement: Supplementary file 1 [file viruses-11-00797-s001.zip › Supplementary Table S1.docx]

Table S1 : List of primers used in this study

| Name | Sequence (5’-3’) |
| --- | --- |
| Viperin | CTTTTGCTGGGAAGCTCTTG |
|  | GTCTCATCTGGCCCTCTCAG |
| OAS1 | CCCAGGAGGTATCAGAAATATGGTTACAT |
|  | AAAATGAAGGAACTGGTCCAGATAACACT |
| OAS3 | CGAGGTCAAGTTTGAAGTCTCC |
|  | AGAGATGATGAAGTCCCGTTGT |
| IL6 | ATGAACTCCTCCTCCACAAGCGC |
|  | GAAGAGCCCTCAGGCTGGACTG |
| TNF-α | CCTGTGAGGAGGACGAACAT |
|  | AGGCCCCAGTTTGAATTCTT |
| GAPDH | TCGGAGTCAACGGAT |
|  | TCGCCCCACTTGATT |
| MMP2 | TCCACTGGATGGAGGAAAAC  AAGCTCTGACCTTTCCAGCA |
| MMP1 | QuantiTect Primer Assay : QT00014581 (Qiagen) |
| MMP8 | QuantiTect Primer Assay : QT00029820 (Qiagen) |
| MMP10 | QuantiTect Primer Assay : QT00001470 (Qiagen) |
| MMP13 | QuantiTect Primer Assay : QT00001764 (Qiagen) |
| MMP14 | QuantiTect Primer Assay : QT00001533 (Qiagen) |
| MMP15 | QuantiTect Primer Assay : QT00014063 (Qiagen) |
|  |  |
