# Supplementary material for: Mayaro Virus Infects Human Chondrocytes and Induces the Expression of Arthritis-Related Genes Associated with Joint Degradation
Source: Viruses. 2019 Aug 29;11(9):797. doi: 10.3390/v11090797 (PMC6783875; doi:10.3390/v11090797)
Supplement: Supplementary file 1 [file viruses-11-00797-s001.zip › Supplementary Table S2.docx]

| **TABLE S2.** Modulation of extracellular matrix gene expression by MAYV infection. | | |  | **TABLE S2.** (Continued) | | |
| --- | --- | --- | --- | --- | --- | --- |
|  | Fold change in gene expression at indicated time postinfection^a^ | |  |  | Fold change in gene expression at indicated time postinfection^a^ | |
|  | 24h | 48h |  |  | 24h | 48h |
| ***ADAMTS1*** | 1.5 | **-2.9** |  | ***ITGB4*** | **-3.2** | **-2.3** |
| ***ADAMTS13*** | **6.5** | **15.2** |  | ***ITGB5*** | **-3.5** | -1.6 |
| ***ADAMTS8*** | **3.7** | **8.5** |  | ***KAL1*** | **-3.7** | **75.5** |
| ***CD44*** | **-26.0** | **-22.0** |  | ***LAMA1*** | **3.4** | **7.2** |
| ***CDH1*** | **-7.9** | **-7.4** |  | ***LAMA2*** | -1.3 | **26.3** |
| ***CLEC3B*** | **5.5** | **119.3** |  | ***LAMA3*** | 1.2 | **343.4** |
| ***CNTN1*** | **5.3** | **10.8** |  | ***LAMB1*** | **-2.0** | **2.4** |
| ***COL11A1*** | **12.9** | **-6.3** |  | ***LAMB3*** | 1.0 | **374.5** |
| ***COL12A1*** | **8.2** | **-97.8** |  | ***LAMC1*** | **-2.6** | **-3.5** |
| ***COL14A1*** | **77.2** | 1.4 |  | ***MMP1*** | **2.1** | **14.3** |
| ***COL15A1*** | **41.7** | **6.1** |  | ***MMP10*** | **35.2** | **62.6** |
| ***COL16A1*** | **-17.7** | **-5.8** |  | ***MMP11*** | **510.7** | **920.5** |
| ***COL1A1*** | **-16.9** | **-223.1** |  | ***MMP12*** | -1.7 | **-3.1** |
| ***COL4A2*** | **-7.3** | **-2.4** |  | ***MMP13*** | **100.6** | **464.5** |
| ***COL5A1*** | **-26.2** | **2.9** |  | ***MMP14*** | **183.1** | **8.4** |
| ***COL6A1*** | **-22.3** | **4.4** |  | ***MMP15*** | **10.7** | **28.2** |
| ***COL6A2*** | **-25.0** | **-4.5** |  | ***MMP16*** | -1.6 | **2.3** |
| ***COL7A1*** | **3.4** | **21.2** |  | ***MMP2*** | **-10.8** | **-18.5** |
| ***COL8A1*** | **-8.4** | **-7.6** |  | ***MMP3*** | **-4.6** | **6.4** |
| ***CTGF*** | -1.9 | **-2.2** |  | ***MMP7*** | **62.6** | **140.7** |
| ***CTNNA1*** | **8.9** | **-15,7** |  | ***MMP8*** | **47.9** | **211. 4** |
| ***CTNNB1*** | **-20.2** | -1.1 |  | ***MMP9*** | **5.1** | **23.1** |
| ***CTNND1*** | **-11.6** | **-9.3** |  | ***NCAM1*** | **3.4** | **159.1** |
| *CTNND2* | ND | ND |  | ***PECAM1*** | **52.4** | **75.3** |
| ***ECM1*** | **4.3** | **23.2** |  | ***SELE*** | **12.2** | **58.6** |
| *FN1* | 1.2 | -1.4 |  | ***SELL*** | **2.5** | **7.1** |
| ***HAS1*** | **9.9** | **20.0** |  | ***SELP*** | **12.8** | **19. 7** |
| ***ICAM1*** | **-3.5** | -1.1 |  | ***SGCE*** | **-41.2** | **-16.4** |
| ***ITGA1*** | **-5.8** | **-2.2** |  | ***SPARC*** | **-16.8** | **-46.7** |
| ***ITGA2*** | **129.0** | **954.6** |  | ***SPG7*** | **6.0** | **322.6** |
| ***ITGA3*** | **77.2** | **377.9** |  | ***SPP1*** | **24.4** | **52.0** |
| ***ITGA4*** | **2.1** | 1.1 |  | ***TGFBI*** | **-14.6** | **-4.0** |
| ***ITGA5*** | -1.2 | **-22.6** |  | ***THBS1*** | **20.6** | **11.7** |
| ***ITGA6*** | **52.6** | **66.2** |  | ***THBS2*** | **13.3** | **26.8** |
| ***ITGA7*** | **19.1** | **627.6** |  | ***THBS3*** | **6.6** | **-7.4** |
| ***ITGA8*** | 1.1 | **54.3** |  | ***TIMP1*** | **29.2** | **4.1** |
| ***ITGAL*** | **4.0** | **4.1** |  | ***TIMP2*** | 1.2 | **11.9** |
| ***ITGAM*** | **24.5** | **80.7** |  | ***TIMP3*** | **29.2** | **77.6** |
| ***ITGAV*** | **-8.3** | **6.4** |  | ***TNC*** | **85.4** | **37.2** |
| ***ITGB1*** | **-14.0** | 1.6 |  | ***VCAM1*** | **6.2** | **-19.8** |
| ***ITGB2*** | 1.2 | **887.6** |  | ***VCAN*** | -1.3 | **-45.1** |
| ***ITGB3*** | -1.7 | **138,0** |  | ^a^ Values represent fold inductions of mRNA copy numbers in infected cells relative to mock-infected cells. Values in bold indicate differentially expressed genes ; ND: not determined. | | |
|  |  |  |  |  |  |  |
